# Prioritizing sleep for healthy work schedules

**DOI:** 10.1186/1880-6805-31-6

**Published:** 2012-03-13

**Authors:** Masaya Takahashi

**Affiliations:** 1 National Institute of Occupational Safety and Health, 6-21-1, Nagao, Tama-ku, Kawasaki 214-8585, Japan

**Keywords:** Alertness, napping, productivity, recovery, sleep

## Abstract

Good sleep is advantageous to the quality of life. Sleep-related benefits are particularly helpful for the working class, since poor or inadequate amounts of sleep degrade work productivity and overall health. This review paper explores the essential role of sleep in healthy work schedules and primarily focuses on the timing of sleep in relation to the work period (that is, before, during and after work). Data from laboratory, field and modeling studies indicate that consistent amounts of sleep prior to work are fundamental to improved performance and alertness in the workplace. In addition, planned naps taken during work maintain appropriate levels of waking function for both daytime and night-time work. Clearly, sufficient sleep after work is vital in promoting recovery from fatigue. Recent data also suggest that the time interval between shifts should be adjusted according to the biological timing of sleep. Although sleep is more likely to be replaced by job and other activities in the real life, research shows that it is worthwhile to revise the work schedules in order to optimize sleep before, sometime during and after the work period. Therefore, we suggest establishing work-sleep balance, similar to work-life balance, as a principle for designing and improving work schedules.

## Introduction

Advances in sleep research have produced a considerable amount of data regarding the role of sleep in all areas of life, including the workforce. Working individuals account for approximately half of the population in most countries, which emphasizes the significance of sleep for this particular group. At a worker level, inadequate sleep leads to a wide range of health disorders [1]. Also, sleep problems are associated with short- and long-term sickness leave [2] and further interfere with return to work following these absences [3,4]. In the long run, poor sleep causes work disability [4] and early retirement [5]. Furthermore, disrupted sleep dose-dependently increases the risk of all-cause mortality in working men [6]. In reference to cause-specific mortality, severely disturbed sleep may be associated with a greater suicide risk, even after accounting for depressive symptoms [6]. This finding is highly relevant to the situations in Japan, with over 30,000 people committing suicide every year since 1998 [7]. Sleep problems are also a safety concern because they are associated with occupational injuries, as shown in both cross-sectional and prospective studies [8,9].

We believe it is important to address sleep-related occupational hazards from not only an individual perspective, but from a workplace perspective. At the workplace level, recent findings highlight the economic burden caused by employees with insufficient and/or poor sleep [10-12]. Although many differences exist among studies, sleep-related costs in the workforce are within a similar monetary range of approximately a few thousand dollars per person per year [10-12]. Importantly, due to the significant costs and the large number of workers affected, employers experience substantial economic damage from sleep-related problems.

Despite the clear disadvantages as listed above, the value of sleep has been somehow neglected. The decline in interest among occupational safety and health experts may occur because they view sleep as a 'private activity' outside of work. Consequently, the attitude towards sleep contrasts with the attitude towards work hours and stressors, even though both are critical to the quality of work.

The present review explores the essential role of sleep in healthy work schedules. This paper particularly focuses on when workers sleep, including before and after an assigned working period, and napping during the work shift.

### Sleep before work: preparation for enhanced productivity

Employees have the responsibility to be at their mental and physical best during a work shift. Achieving adequate amounts of sleep prior to work is a key factor contributing to productivity. In this context, the prior sleep wake model proposed by Dawson and McCulloch provides a simple, yet effective framework [13]. This model requires a certain minimum duration (X and Y hours, respectively) of sleep during the 24-hour and 48-hour period prior to work, in order to prevent errors and accidents during wakefulness from awakening to end of work. Currently, X and Y are assumed to be 5 and 12 hours, respectively.

**Figure 1:**
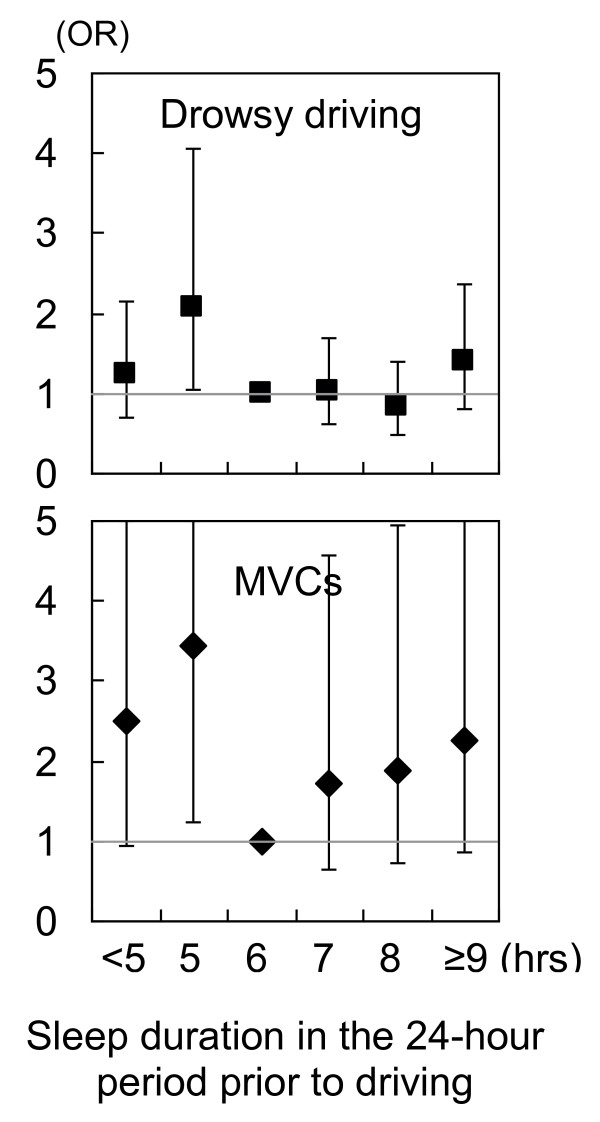
**Reported incidence of drowsy driving and motor vehicle crashes associated with sleep duration in the 24-hour period prior to driving among truck drivers **[18]. Data are odds ratios (ORs) adjusted for major confounding factors. Error bars represent 95% confidence intervals (CIs). For the motor vehicle crashes, the upper 95% CI was 6.61 in the < 5-hour sleep, 9.68 in the 5-hour sleep, and 5.83 in the ≥ 9-hour sleep groups.

The validity of the proposed model has been tested across multiple work settings. For example, fatigue-related accidents by truck drivers were accurately predicted by X = 6.5 and Y = 8 hours [14]. Data among train industry employees (for example, train drivers, controllers and guards) showed that extreme tiredness or exhaustion was significantly associated with sleep duration 24 hours prior to work, shift length, night shifts and workload, and that such symptoms were assumed to be reduced by 12% if the sleep was increased by one hour during that 24-hour period [15]. In airline crews, poor operational performance and increased errors were significantly associated with less than 6 hours of sleep in the 24 hours prior to work [16]. Similarly, miners who took less than 6 hours of sleep in the prior 24-hour period performed a psychomotor vigilance task (PVT) significantly slower than miners who had adequate sleep [17]. We also used the prior sleep wake model to analyze truck driver data and found that drowsy driving and/or motor vehicle crashes increased when drivers had 5 or fewer hours of sleep in the 24-hour period prior to the start of driving (Figure 1) [18].

Many workers experience chronic partial sleep deprivation over weekdays. Experimental evidence indicates that repeated partial sleep deprivation, even achieving 5 to 6 hours of night's sleep, causes a gradual impairment in neurobehavioral performance each day [19,20]. Extending sleep duration prior to partial sleep deprivation may counteract the subsequent impairments in performance. Indeed, a recent study showed that 10-hour time in bed (TIB) (approximately 8-hour total sleep time [TST]) for one week prior to seven days of sleep restriction (3 hours per night) yielded better PVT performance and electroencephalogram-defined alertness than spending 7-hours TIB (approximately 6-hour TST) the week before sleep restriction [21]. Moreover, the aforementioned sleep extension facilitated recovery in performance and alertness following sleep restriction. These findings suggest that banking extra sleep may be an effective coping strategy to ensure work performance and to improve the recovery when one anticipates a period of by chronic sleep deprivation.

Taken together, research demonstrates that acquiring an appropriate amount of sleep is a fundamental component to work productivity. We are not allowed to go to work while intoxicated; physiologically, skipping sleep has similar influences on the brain and body as drinking alcohol [22]. Consequently, we should not be allowed to work when we are sleep deprived. More attention should be directed to achieving appropriate and sufficient amounts of sleep prior to one's work shift.

### Naps during work: maintenance of on-the-job performance

Generally, employees are not entitled to take naps during their work periods. Napping is often confused with dosing off, which tends to be viewed as a counterproductive behavior. However, planned naps, if taken during working hours, are recognized as a promising technique in maintaining job performance and alertness [23-26]. Particularly, a brief (that is, 15 to 20 minutes) nap improves waking function [23,27,28] and is ideal for employees, given the limited opportunity for napping in the workplace. Recent neuropsychological research supports this idea and has indicated that the nap-related gains in learning and memory may be potential mechanisms for improving cognitive function [29,30].

In addition to the neurobehavioral benefits, planned napping may have favorable impacts on worker health. Based on results from a cohort study of over 23,000 participants, men who took 30-minute naps occasionally or napped at least three times per week showed a 50% decrease in their risk of coronary-related mortality (hazard ratio 0.51, 95% confidence interval 0.32 to 0.83) [31]. Interestingly, this association was stronger for working men at enrollment (hazard ratio 0.36, 95% confidence interval 0.16 to 0.77) than for non-working men at enrollment (hazard ratio 0.64, 95% confidence interval 0.33 to 1.21). Although the specific underlying mechanisms for these findings remain unclear, several immunological studies have revealed that while one night's sleep deprivation increased a proinflammatory cytokine (IL-6), a 2-hour nap (2 p.m. to 4 p.m.) significantly reduced this effect. Moreover, the IL-6 level continually decreased following the nap period [32]. Similarly, sleep restriction of only 2 hours a night decreased immunological parameters, while the addition of a 30-minute daytime nap facilitated immunological recovery [33].

**Figure 2:**
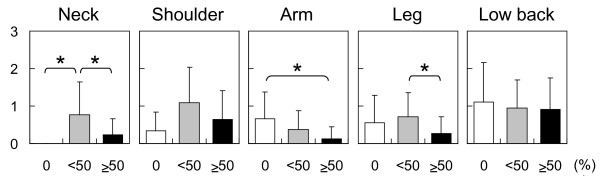
**Pain scores in the neck, shoulder, arm, leg, and low back according to frequency of night-shift naps during the previous month **[37]. The nap frequency was quantified within a 0% to 100% range (0% = no nap at all, 100% = nap taken during every night shift). Musculoskeletal pain at the current time was rated on a four-point scale (0 = almost no pain, 1 = mild, 2 = moderate, 3 = severe). Error bars represent standard deviations. *P < 0.05.

Appropriate use of napping is thought to be more beneficial for night and shift workers [23]. Indeed, when a nap schedule was integrated into the work shift of medical interns, those that napped slept longer while on-call and experienced less fatigue compared to interns that did not have a nap schedule [34]. Concurrently, research with health care workers showed that a 30-minute opportunity for napping in the middle of a night shift (2 a.m. to 3 a.m.) improved PVT performance and subjective sleepiness compared with no nap [35]. Findings from qualitative research with hospital nurses suggest that naps prove restorative during night shifts and that naps are necessary to achieve a high quality of care [36]. Many health care professionals who work at night and shift-work suffer from musculoskeletal disorders. Recent findings among nursing home caregivers indicate that taking a nap at least once every two night shifts was significantly associated with reduced pain in the neck, arm and leg, though not low back pain (Figure 2) [37]. These data imply that protecting the time for night-shift napping may also be effective in preventing work-related musculoskeletal injuries.

Importantly, naps are beneficial for a variety of workers, in addition to those in the health care industry. That is, international airline pilots take in-flight naps according to their perceived level of fatigue, which reiterates the use of naps as a preventive measure [38]. In addition, night shift air traffic controllers given a 40-minute nap opportunity displayed better PVT performance and objective measures of alertness compared to controllers in the no-nap condition [39]. The similar favorable effects of a nap have been recognized for car driving [40].

In recent years, there has been a growing interest in the possible link between shift work, night shifts in particular, and cancer [41]. This concern is corroborated by the fact that the International Agency for Research on Cancer categorized 'shift work that involves circadian disruption' as probably carcinogenic to humans (Group 2A) [42]. Exposure to environmental light during the night shift suppresses melatonin, which is thought to be responsible for an increased cancer risk [43,44]. If so, taking a night-shift nap in a darkened room may exert a protective role against cancer through the reduction in light exposure; however, this hypothesis has yet to be examined.

The above arguments support the active use of naps during daytime and shift work periods. Sleep inertia (SI), transient decreases in performance and alertness that occur immediately after awakening, is sometimes regarded as a barrier to worksite napping [36]. However, we are able to minimize the negative effects of SI by providing either additional time after the nap, or by adjusting the nap duration, in order to restore the central nervous system function [45]. Therefore, although the effects of SI cannot be ignored, the value of on-the-job napping is important to overall function and should be considered in the workplace.

### Sleep after work: recovering from fatigue

It is undisputable that sleep plays a vital role in one's recovery following a work period [46]. Chronic partial sleep deprivation studies emphasize the need for adequate sleep each and every night [19,20]. Unfortunately, the reality is that work and other activities are often completed at the expense of sleep. Several worksites in Japan have implemented a 'no-overtime day' in the middle of the week (for example, on Wednesday). Nevertheless, no empirical data are available regarding its effects on health, sleep or performance.

As it may be difficult to get adequate sleep every night, the second best opportunity for taking a sufficient sleep may be on weekends. We tested the hypothesis that extended sleep during weekends would improve performance and alertness during the subsequent weekdays in daytime workers, specifically in those with habitually limited sleep (6 hours or less) [47]. One concern related to sleep extension on weekends is the 'Blue-Monday effects', which is the impaired waking function during the first half of the subsequent week due to delays in circadian phase by sleeping in on the weekend [48]. To minimize these effects, participants in the intervention condition were instructed to stay in bed for ≥ 8 hours between 10 p.m. and 9 a.m. and were prohibited from taking a daytime nap on weekends (Friday, Saturday and Sunday). Participants in the control condition were asked to keep their normal weekend sleep-wake pattern.

**Figure 3:**
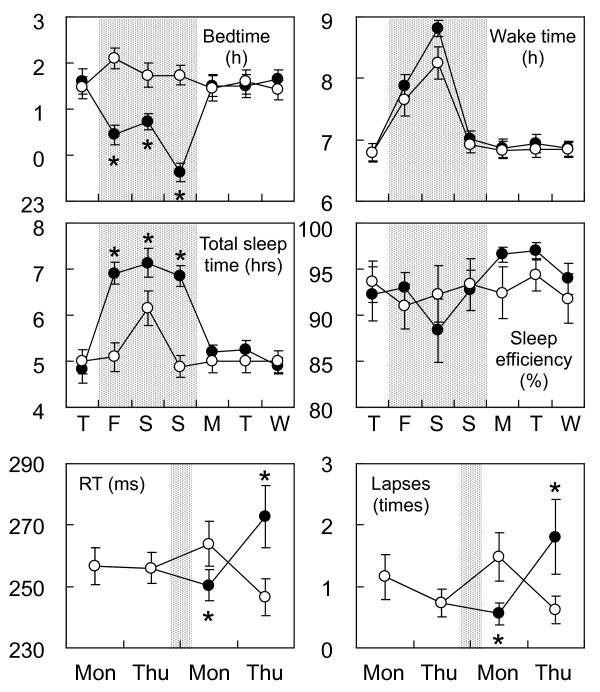
**Actigraphic sleep parameters before, during and after weekend sleep intervention and psychomotor vigilance task performance before and after the intervention **[47]. Shared area indicates the period of the intervention. Filled circles represent weekend sleep intervention condition and open circles represent the habitual sleep condition as control. Lapses were defined as the number of reaction times (RTs) exceeding 500 ms. Error bars represent standard errors. *P < 0.05 from the control condition.

As depicted in Figure 3 (bedtime, wake time and TST), participants successfully adhered to the study, with the high quality of sleep measured by sleep efficiency. As expected, PVT performance on the Monday following the intervention condition was significantly better than the performance in the control condition. Interestingly, the opposite pattern was observed on Thursday. The explanations for these results are not yet clear, but an important factor may be the return to the habitual shorter sleep duration (approximately 5 hours TST) during the post-intervention week. In other words, the benefits of weekend sleep extension might have been maintained, if the participants had continued to achieve sufficient sleep during the week.

Particular care needs to be given to post-shift night sleep in order to support optimal recovery [49]. A long interval from a shift to the next shift is thus required. This feature may be critical to recovery, as sleep and health indicators have improved with the change from a fast, forward-rotating three-shift schedule (two days on each shift followed by four days off) to a slow, backward-rotating schedule with intermediate days off between shifts (three days on each shift followed by three days off prior to the next shift) [50].

**Figure 4:**
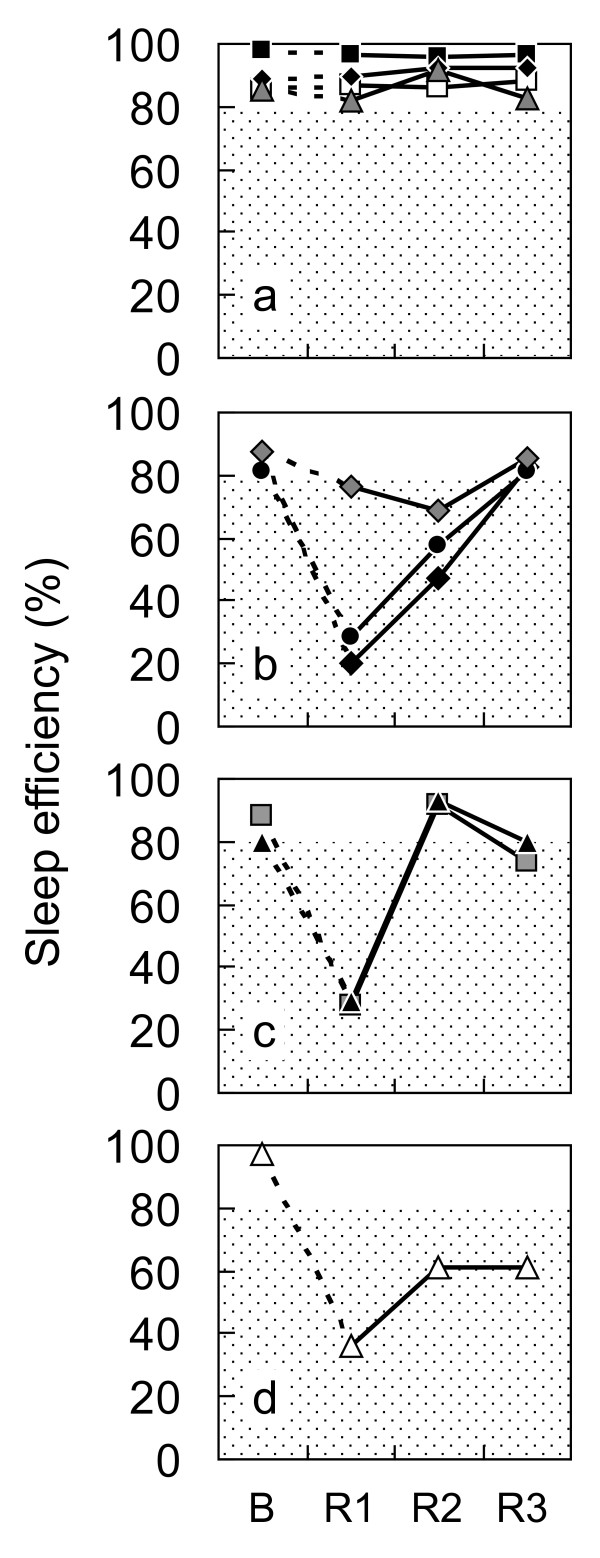
**Recovery of sleep after simulation over four successive night shifts **[53]. **(a) **Rapid (n = 4), **(b) **slow (n = 3), **(c) **pseudo (n = 2), and **(d) **incomplete (n = 1) recovery groups. B: baseline night; R: recovery night.

Another important variable to consider is the individual differences in response to displacement of sleep disruption or sleep restriction [51,52]. Preliminary results assessing sleep recovery following four successive night shifts showed that there are at least four types of recovery: rapid, slow, pseudo and incomplete types (Figure 4) [53]. The recovery types were not associated with morningness-eveningness, the quality of daytime sleep during the night shift period, PVT performance or subjective sleepiness during the simulated night shifts. Instead, the inter-individual differences were associated with the participants' sleep habits, more specifically the timing of sleep (bedtime and the variability of wake time).

The latest findings demonstrate that not only is the length of shift interval (or days off) important, but that circadian timing should also be considered [54]. The same interval length between shifts does not necessarily exert the same recuperative power in regards to the time of day when the shift interval occurs. Although the current principles of shift work and working time recommend a certain length of rest (for example, 11 successive hours) [55,56], additional revisions can be made to accommodate the circadian viewpoint [49].

In summary, work schedules should be designed to incorporate a sufficient time interval after every work period to promote sleep recovery. Moreover, the timing and duration of the rest interval need to be adjusted to account for shift requirements and circadian rhythms in order to ensure optimal recuperation.

### Future directions

**Figure 5:**
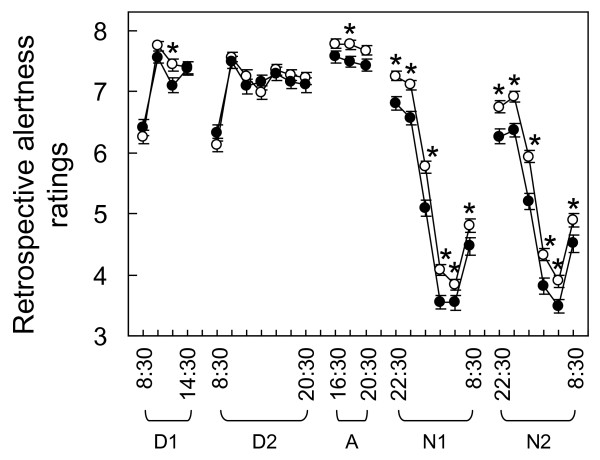
**Retrospectively rated alertness during each shift for operators reporting good (open circle) and poor (filled circle) perception of adaptation to shift work **[57]. Higher scores indicate greater alertness: 7 = alert, 5 = neither alert nor sleepy, 3 = sleepy (but not fighting sleep). Error bars represent standard errors. *P < 0.05 (adjusted for age). A: afternoon shift; D: day shift; N: night shift.

In a perfect world, employees would be very well prepared for demanding schedules such as shift work and long work hours. However, even in this ideal view, it is likely that some portion of employees will still experience health risks. The important point is to detect sub-optimal health conditions and then to take appropriate action. In this regard, assessing sleep and alertness may provide useful information. In the previous study, nuclear power plant operators who displayed poor adaptation to their assigned work shift showed elevated sleep disturbances across sleep periods and unfavorable results on various outcomes compared to operators in the well-adapted group (Table 1) [57]. Moreover, poor-adaptors reported decreased alertness during shifts, especially night shifts, compared to their well-adapted colleagues (Figure 5).

**Table 1:** Sleep, health, and job outcomes for groups of nuclear power plant operators that perceive good or poor adaptation to shift work [57]

	Perceived adaptation				
		
	Good (n = 250)		Poor (n = 358)		*P* ^a^
		
	Mean	(SD)	Mean	(SD)	
Fit to job content (%)	80.3	(13.0)	59.4	(19.6)	< 0.001
Eveningness (currently)	3.7	(1.0)	3.2	(1.1)	< 0.001
Social and family disruption	16.1	(6.4)	20.0	(5.6)	< 0.001
Chronic fatigue	89.5	(21.9)	103.7	(20.9)	< 0.001
Shift system advantages	5.4	(2.1)	5.0	(1.7)	0.023
Shift work locus of control	107.2	(15.2)	99.1	(16.1)	< 0.001
Psychological wellbeing (GHQ-12)	11.6	(3.5)	13.6	(4.2)	< 0.001
Daytime sleepiness (ESS)	6.7	(2.8)	7.1	(2.9)	0.033
Sleep disturbance					
Day shift 1	20.8	(6.2)	22.3	(6.0)	0.002
Day shift 2	20.8	(5.9)	22.2	(5.7)	0.005
Afternoon shift	17.6	(5.9)	19.3	(5.9)	0.007
Night shift 1					
between afternoon shift and night shift 1 (night)	17.8	(6.2)	19.5	(6.1)	0.004
Before night shift 1 (daytime)	22.8	(7.5)	27.7	(6.8)	< 0.001
Night shift 2					
between night shift 1 and night shift 2 (daytime)	22.7	(7.2)	26.3	(7.4)	< 0.001
after night shift 2 (daytime)	22.0	(6.3)	24.9	(6.7)	< 0.001
on the first day off (night)	16.7	(6.3)	18.8	(6.3)	0.001
Napping during night shifts (hours)					
Night shift 1	1.5	(0.9)	1.3	(0.9)	0.001
Night shift 2	1.8	(1.5)	1.5	(1.3)	0.001

^a^Adjusted for age. Fit to job content was assessed by the question 'How well do you fit the current job content?' on a 0 to 100% scale in 10% units. Higher scores for sleep disturbance indicate more disturbances. ESS: Epworth Sleepiness Scale; GHQ-12: 12-item General Health Questionnaire.

Physiologically, melatonin (for example, dim light melatonin onset) and body temperature may become candidates for health evaluations assessing the response to work schedules. These parameters, however, are easily masked by environmental conditions [58]. Additionally, it is quite difficult to establish normal levels under continuously perturbed work, sleep and light-dark cycle. In contrast, profiling clock genes is expected to be a breakthrough method for assessing work schedule-related damages to health [59,60]. More research is needed to determine its validity and feasibility.

Given the large variability in recent work schedules, biomathematical modeling may be a valuable tool to predict how sleep and performance are maintained [58,61]. Enhanced communication and collaboration is needed between laboratory experiments and field studies to address the improvements and sophistication of the given models.

Finally, a large portion of sleep research has focused on the negative consequences of sleep disruption or restriction, such as impaired health and decreased job performance. To better disseminate the importance of sleep to employers and many stakeholders, research should focus more on the benefits of obtaining adequate sleep. Several lines of evidence did demonstrate that sleep should be optimized to make employees more healthy and creative [62-64].

## Conclusions

Numerous research findings so far support the idea that that inadequate sleep is detrimental to both workers and the workplaces. Although further studies are needed to establish a specific causal relationship, action should be taken to protect employees, employers and stakeholders against sleep-related disadvantages. Work schedules affect every aspect of working life; improper scheduling may result in sleep restriction and/or disturbance, which could then lead to dire consequences for everyone. However, work schedules can be modified to ensure that workers are able to get optimal levels of sleep before, sometime during, and after the work period. Although work-life balance has received much attention, here we propose that work-sleep balance, through the creation of healthy work schedules, is equally essential for improved working life.

## Competing interests

The authors declare that they have no competing interests.
